# Supplementary material for: Astrocyte plasticity in mice ensures continued endfoot coverage of cerebral blood vessels following injury and declines with age
Source: Nat Commun. 2022 Apr 4;13:1794. doi: 10.1038/s41467-022-29475-2 (PMC8980042; doi:10.1038/s41467-022-29475-2)
Supplement: Supplementary file 1 — Supplementary Information [file 41467_2022_29475_MOESM1_ESM.pdf]

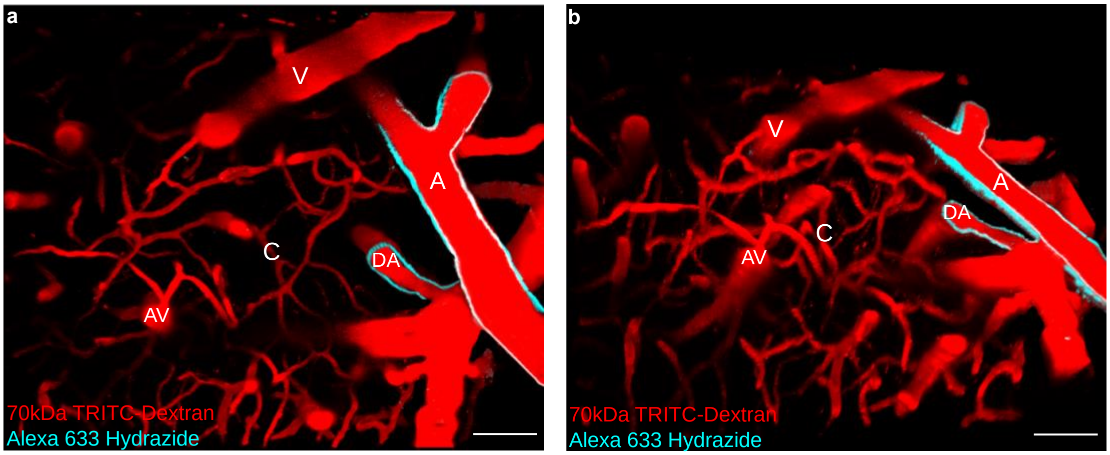

Supplementary Figure 1-Alexa 633 hydrazide positively selects for arterioles- In order to determine which vessel type we were ablating astrocytes at, we employed the use of Alexa 633 hydrazide (cyan) to: positively select for arterioles and penetrating arterioles; exclude vessels (shown in red) based on size; and negatively select against venules, ascending venules, and capillaries (<10 µm). Volumetric reconstruction showing **a)** a dorsal view of all zones of the vascular tree and **b)** a dorsolateral view of the same field. Scale bar=25 µm. A- arteriole, DA- descending arteriole, V- venule, AV- ascending venule, C- capillary. n=30 fields across 10 mice. Blood vessels are shown in red.

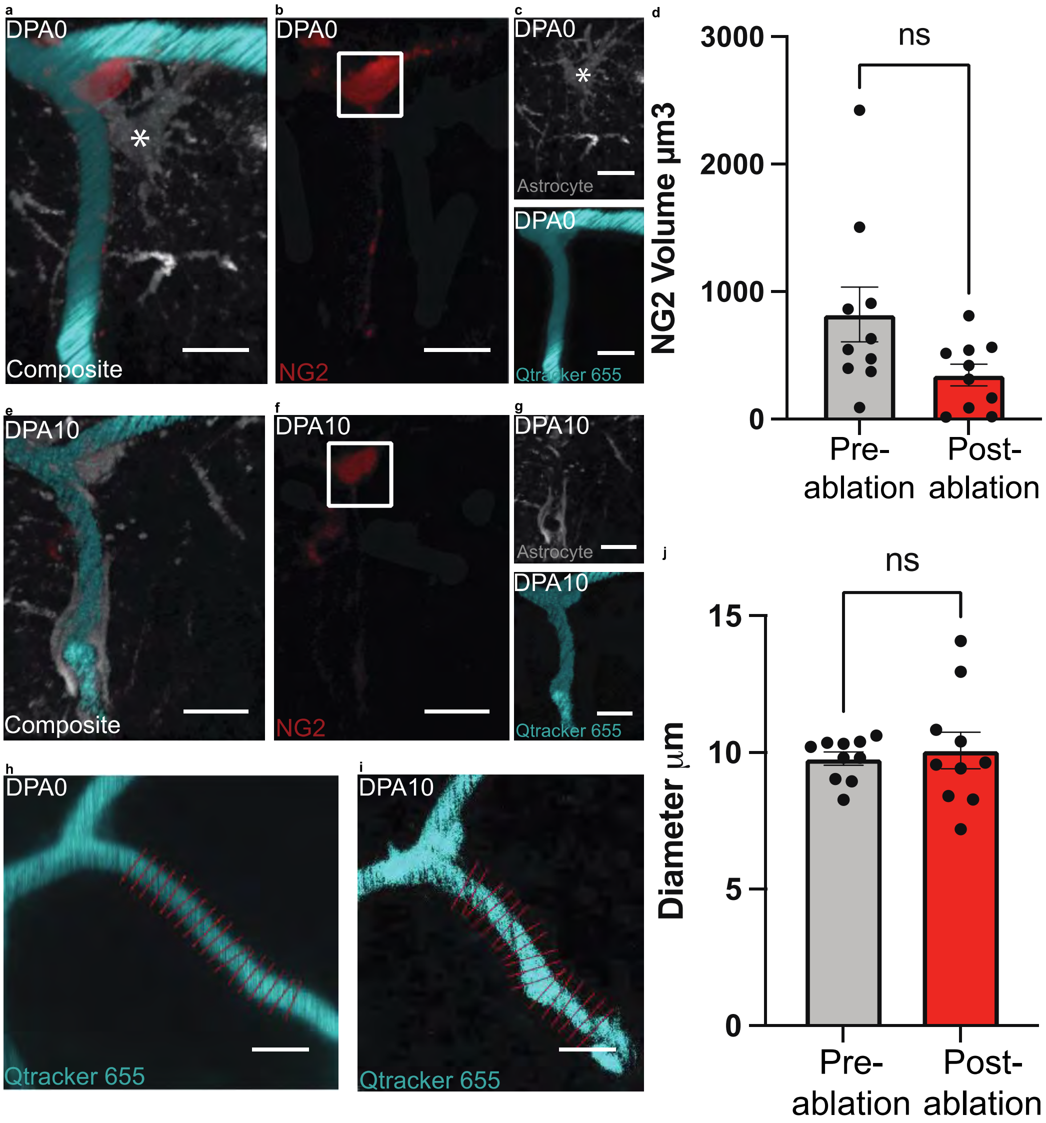

Supplementary Figure 2- Focal ablation of astrocytes does not significantly perturb NG2+ pericyte volume or capillary diameter- In order to determine if focal astrocyte ablation would impact NG2+ pericyte volume and capillary diameter post-ablation, we 2Phatal ablated astrocytes (grey) making contact with NG2+ pericytes (red) on capillaries (cyan) and compared both NG2 volume and capillary diameter post-ablation relative to baseline. **a)** Volumetric reconstruction showing a pericyte-covered capillary with an interacting astrocyte. The asterisk indicates the ablated astrocyte. n=10 NG2+ pericyte somas across 3 mice. **b)** and **c)** Volumetric reconstructions of the same field as in a) with the components isolated, including b) the NG2+ pericyte, c) **(top)** the eGFP+ astrocyte, and **(bottom)** the Qtracker 655-labeled capillary. The square in b) indicates the NG2+ pericyte soma measured. n=10 NG2+ pericyte somas across 3 mice. **d)** Analysis of NG2+ pericyte volume (µm^3) pre- versus post-ablation. Two-tailed paired t-test, $p<0.0599$, data is not significant. n=10 NG2+ pericyte somas across 3 mice. **e)** Volumetric reconstruction showing the

same field at 10 days post-ablation. n=10 NG2+ pericyte somas across 3 mice. **f)** and **g)** Volumetric reconstruction of the same field at dpa10 with the components isolated, including f) the NG2+ pericyte, g) **(top)** the eGFP+ astrocyte, and **(bottom)** the Qtracker 655-labeled capillary. The square in f) indicates the NG2+ pericyte soma measured. n=10 NG2+ pericyte somas across 3 mice. **h)** and **i)** Maximum intensity projections used to measure capillary diameter at h) baseline or i) 10 days post-ablation. n=10 capillaries across 3 mice. VasoMetrics was used to assess diameter: the red lines in both images represent the cross-sectional lines applied by the program to determine mean capillary diameter at both time points. **j)** Capillary diameter pre- versus post-ablation. Two-tailed paired t-test, data is not significant. n=10 capillaries across 3 mice. Scale bar = 10μm in all images. Data are presented as mean values ± SEM.

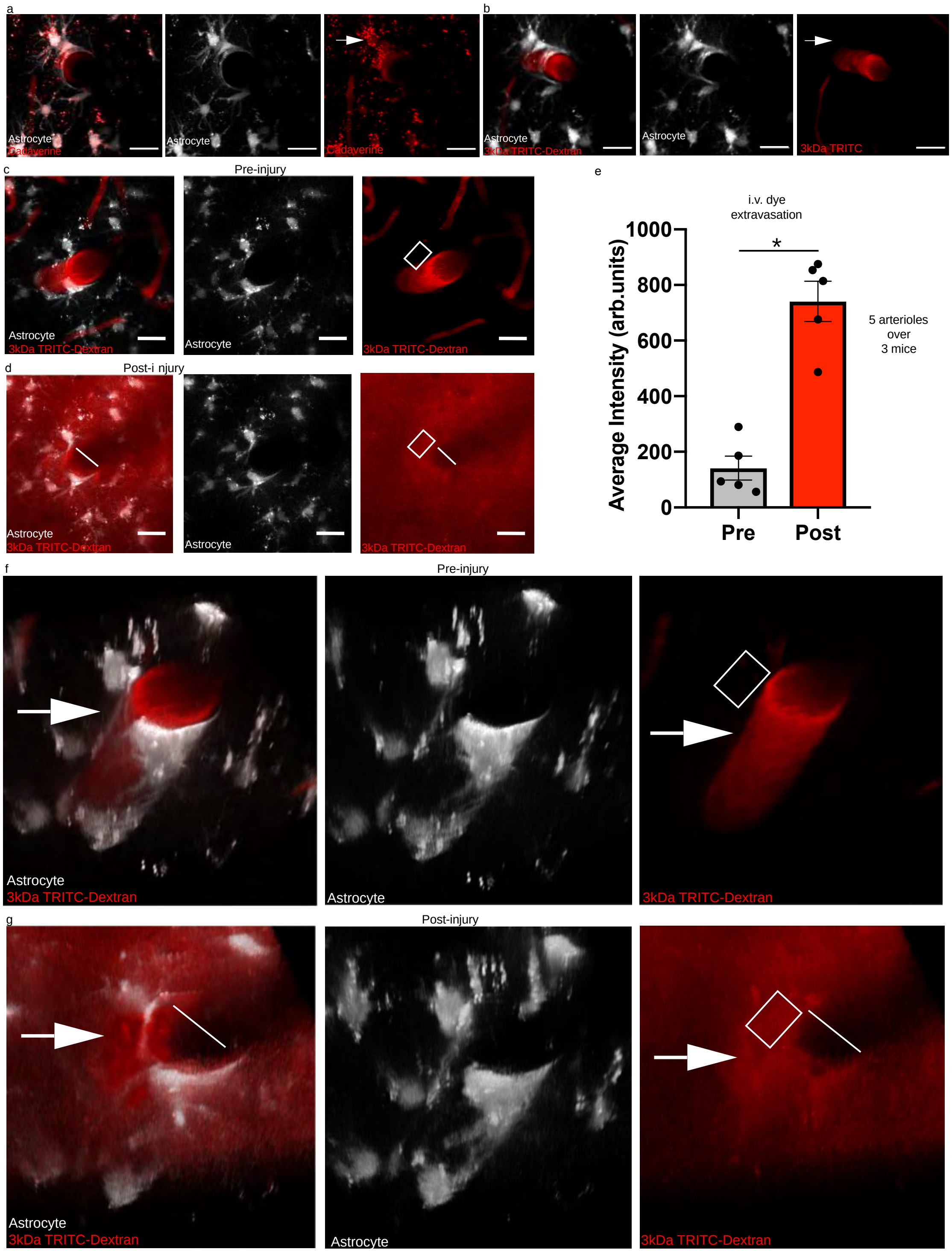

Supplementary Figure 3-Extravasation of 3kDa TRITC can be reliably detected *in vivo*- To measure breaches in BBB integrity, we employed the use of small molecular weight dyes and determined through laser-induced vessel injury that they can be reliably detected *in vivo*. **a)**

Maximum intensity projections showing 967 Da Cadaverine Alexa Fluor 555 (ThermoFisher catalogue number A-30677, shown in red) extravasation at baseline in Aldh1l1-eGFP mice (astrocytes shown in grey) (**left**, **middle**, and **right**). n=5 arterioles across 3 mice **b)** Maximum intensity projections showing that 3kDa TRITC (red) does not extravasate at baseline in Aldh1l1-eGFP mice (astrocytes in grey) (**left**, **middle**, and **right**). Scale bar in a-b=20 µm. n=5 arterioles across 3 mice **c)** Maximum intensity projections showing a penetrating arteriole at baseline without apparent 3kDa TRITC extravasation relative as opposed to **d)** obvious 3kDa TRITC dye extravasation following vessel injury. n=5 arterioles across 3 mice. The white line indicates where the laser scanned across the vessel lumen, and the white box indicates where measurements were taken. Scale bar in c and d=20 µm. **e)** Comparison of the average intensity of 3kDa TRITC extravasation pre-vessel injury relative to post-injury. n=5 arterioles across 3 mice, Two-tailed paired t-test, $p<0.0021$. **f-g)** Volumetric reconstructions showing a dorsolateral view of the same images displayed in c and d. Data are presented as mean values ± SEM.

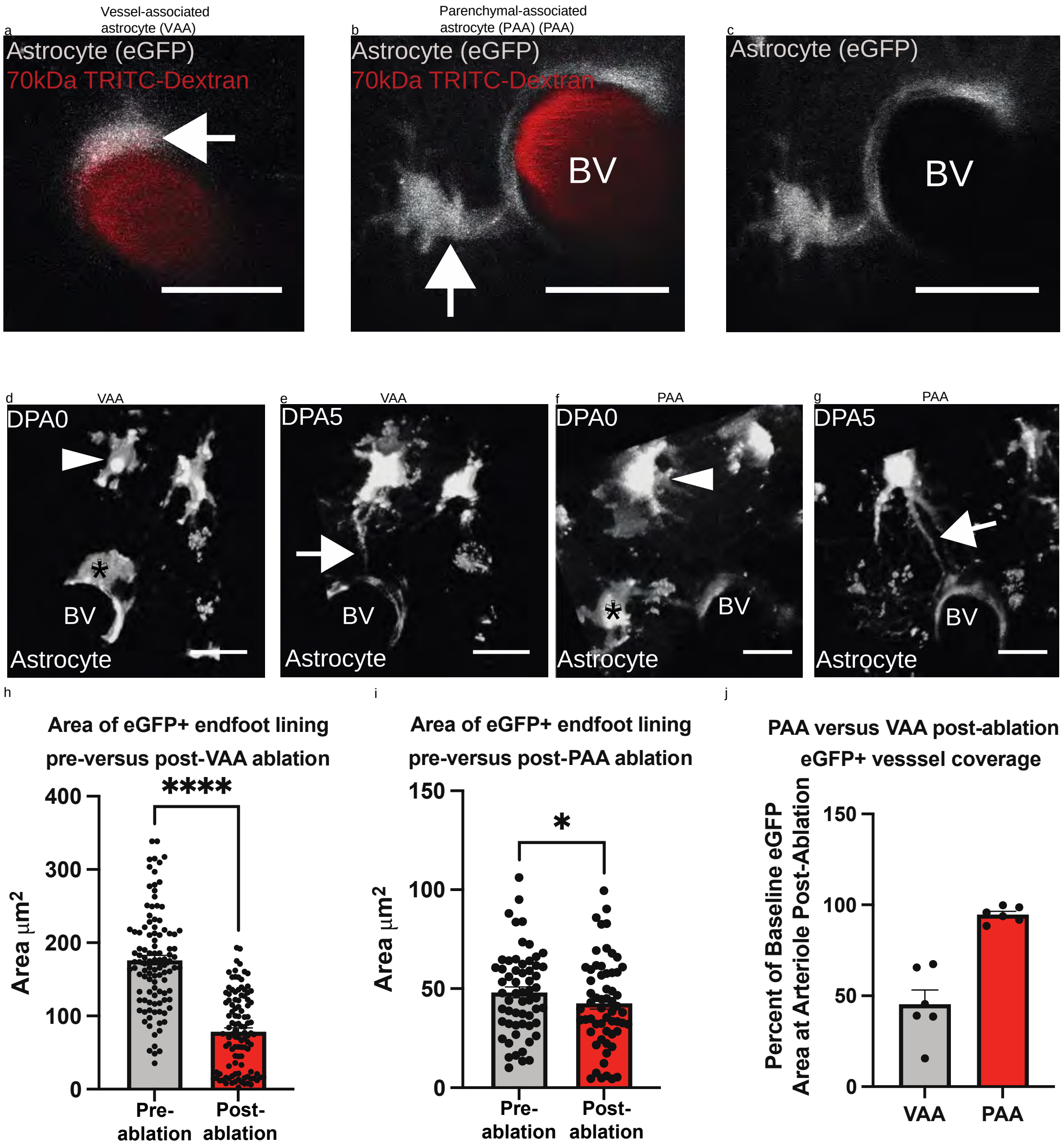

Supplementary Figure 4-The ablation of one astrocyte initiates a replacement response, albeit with significantly reduced post-ablation area of eGFP+ endfoot vessel lining- a) Maximum intensity projection showing a VAA in grey and its relationship with a TRITC-Dextran injected blood vessel in red. n= 40 astrocytes across 5 mice. **b)** Maximum intensity projection showing a PAA in grey and its relationship with a TRITC-Dextran injected blood vessel, labeled BV. **c)** The PAA isolated from b) to show its relationship with the blood vessel (BV). n=40 astrocytes across 5 mice **d)** Volumetric reconstruction showing the dorsolateral view of astrocytes and their association with a blood vessel (BV) at baseline. The asterisk indicates the ablated VAA astrocyte, and the arrowhead the future replacement astrocyte. **e)** Volumetric reconstruction showing the dorsolateral view of the same field from d) at five days post-ablation (DPA5). The arrow demarcates a replacement process from the replacement astrocyte. n=6 astrocytes across 4

mice **f)** Volumetric reconstruction showing the dorsolateral view of astrocytes and their association with a blood vessel (BV) at baseline. The asterisk indicates the ablated PAA astrocyte, and the arrowhead the future replacement astrocyte. **g)** Volumetric reconstruction showing the dorsolateral view of the same field from f) but at DPA5. The arrow demarcates a replacement process from the replacement astrocyte. n=14 fields of view across 7 mice. **h)** Average area of eGFP+ endfoot lining around arterioles at ablated VAA locations pre- versus post-ablation. Two-tailed paired end Wilcoxin matched-pairs signed rank t-test, $p=0.0001$, n=107 optical sections across 4 mice. **i)** Average area of eGFP+ endfoot lining around arterioles at ablated PAA locations pre-versus post-ablation. Two-tailed paired end t-test, $p<0.0133$, n=59 optical sections/4 mice. **j)** Averaged post-ablation eGFP+ endfoot vessel lining area expressed as a percentage of baseline for both single VAA and PAA ablations. n= focal VAA/PAA ablations at 6 arterioles across 4 mice. Note that the data points in h) and i) are averaged for j). LUTS have been adjusted to emphasize the replacement astrocytes and associated processes. Scale bar in all images = 20µm. Data are presented as mean values ± SEM.

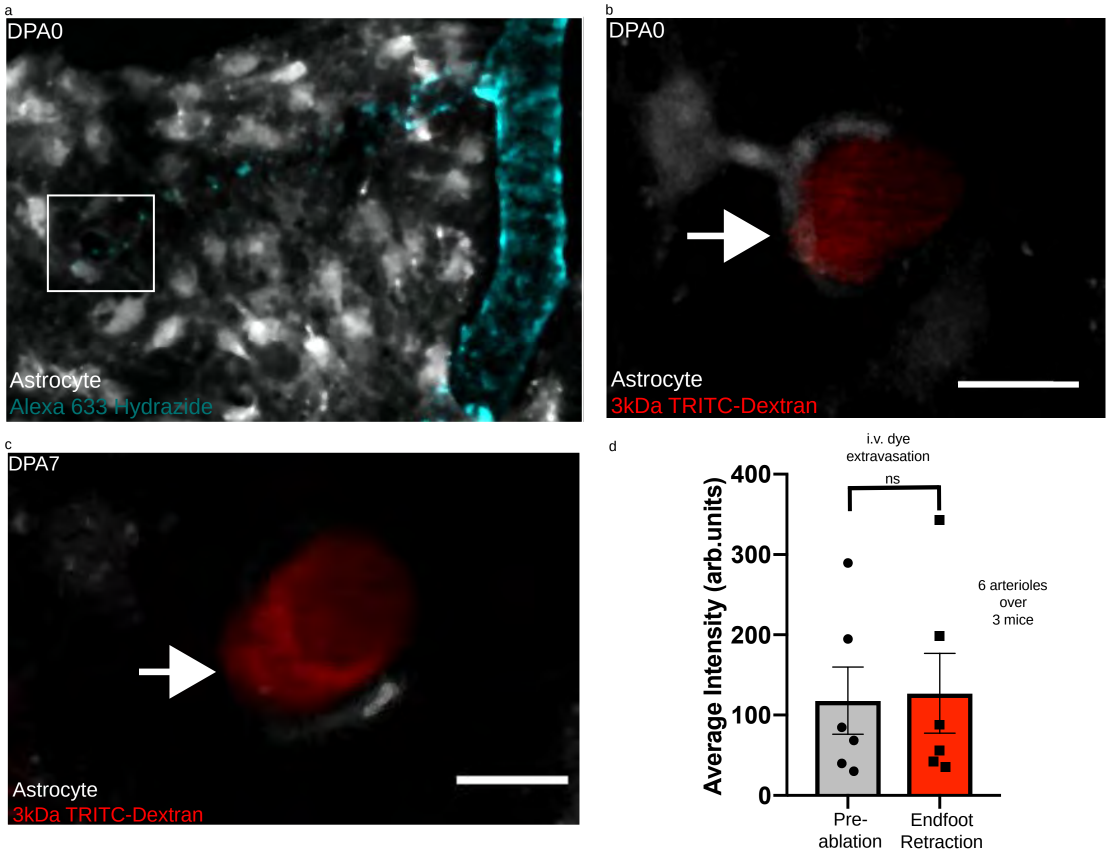

Supplementary Figure 5-Blood-brain barrier integrity is not compromised in 12-month-old mice a) Maximum intensity projection showing an Alexa-633 hydrazide (cyan) positive arteriole in a 12-month-old Aldh1l1-eGFP mouse. n=6 arterioles across 3 mice (astrocytes in grey). White square indicates a penetrating portion of a branch from that arteriole, shown at **b)** a higher magnification at baseline (arteriole in red), and **c)** the same magnification at the exact moment of endfoot retraction on dpa7. The arrow demarcates the absence of the ablated astrocyte and the region of vasculature devoid of endfoot contact. Scale bar in b and c = 10 µm. n=6 arterioles across 3 mice. **d)** Comparison of the average intensity of 3kDa TRITC at baseline, prior to astrocyte ablation, relative to post-ablation. n=6 arterioles across 3 mice, Two-tailed paired t-test, $p<0.3785$. Data are presented as mean values ± SEM.

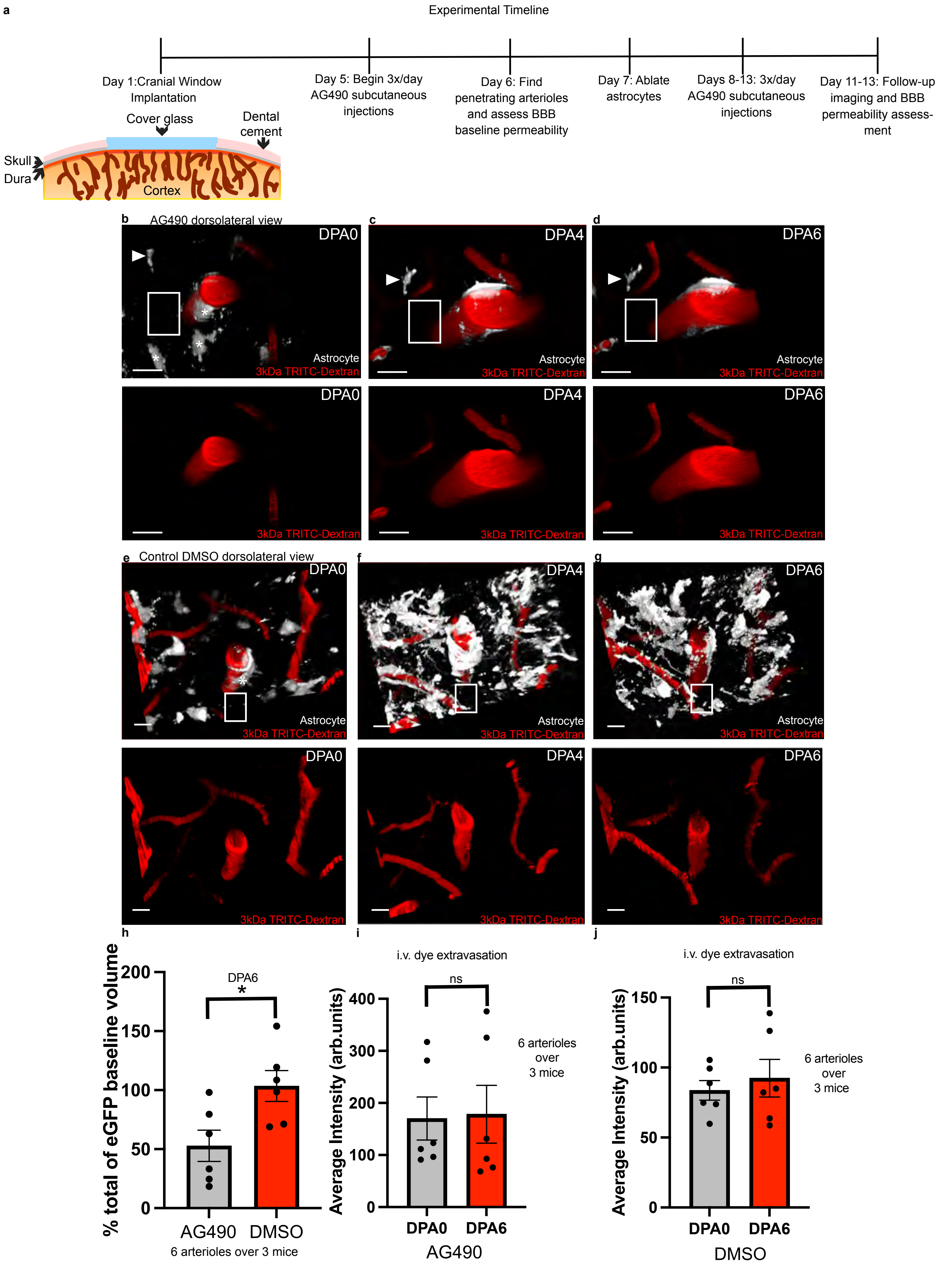

a
Experimental Timeline
Day 1:Cranial Window Implantation
Day 5: Begin 3x/day AG490 subcutaneous injections
Day 6: Find penetrating arterioles and assess BBB baseline permeability
Day 7: Ablate astrocytes
Days 8-13: 3x/day AG490 subcutaneous injections
Day 11-13: Follow-up imaging and BBB permeability assess-ment
Cover glass
Dental cement
Skull
Dura
Cortex
b AG490 dorsolateral view
c
d
DPA0
DPA4
DPA6
Astrocyte
3kDa TRITC-Dextran
e Control DMSO dorsolateral view
f
g
h
DPA6
*
% total of eGFP baseline volume
200
150
100
50
0
AG490
DMSO
6 arterioles over 3 mice
i
i.v. dye extravasation
ns
Average Intensity (arb.units)
400
300
200
100
0
DPA0
DPA6
AG490
6 arterioles over 3 mice
j
i.v. dye extravasation
ns
Average Intensity (arb.units)
150
100
50
0
DPA0
DPA6
DMSO
6 arterioles over 3 mice

Supplementary Figure 6-Significant reductions in astrocyte volume at penetrating arterioles post-ablation is sufficient to maintain BBB integrity- In order to determine how BBB integrity would be impacted following a significant reduction in endfoot coverage post-astrocyte ablation, we adopted the experimental timeline outlined in **a)**. Following cranial window implantation, mice received 3 doses of AG490 daily beginning at day five post-operation. This injection continued through post-operation day 13. After ablating astrocytes (shown in grey, blood vessels are in red) on day seven post-operation, follow-up imaging would occur to assess endfoot plasticity volume for three days (days four to six post-ablation). **b)** Volumetric reconstruction at baseline depicting a dorsolateral view of the astrocyte-vascular landscape **(top)** or just vascular landscape **(bottom)** in AG490-injected mice. **c)** Same field as b, but day 4 post-ablation, and **d)** at day 6 post-ablation. n=6 arterioles across 3 mice. Arrow-head indicates a reference astrocyte. Asterisks indicate ablated astrocytes. Scale bar in b-d= 20 µm. **e)** Volumetric reconstruction at baseline depicting a dorsolateral view of the astrocyte-vascular landscape **(top)** or just vascular landscape **(bottom)** in DMSO control-injected mice. Asterisk indicates the ablated astrocyte. **f)** Same field as e, but at day 4 post-ablation, and **g)** at day 6 post-ablation. n=6 arterioles across 3 mice. **h)** Quantification of the percent change in astrocyte volume at dpa6 relative to dpa0 along arteriole locations where astrocytes were ablated. n=6 arterioles over 3 mice. Two-tailed unpaired t-test, $p< 0.0213$. **i)** Average intensity of 3kDa TRITC dye extravasation along arteriole locations where astrocytes were ablated at dpa6, relative to baseline in AG490-injected mice. n=6 arterioles across 3 mice. Two-tailed paired t-test. $P<0.4104$. **j)** Average intensity of 3kDa TRITC dye extravasation along arteriole locations where astrocytes were ablated at dpa6, relative to baseline in DMSO-injected control mice. n=6 arterioles across 3 mice. Two-tailed paired t-test. $p< 0.5861$. White boxes in b-g represent locations of ROI placement for BBB measurements. Scale bar in e-g=10 µm. Data are presented as mean values ± SEM.

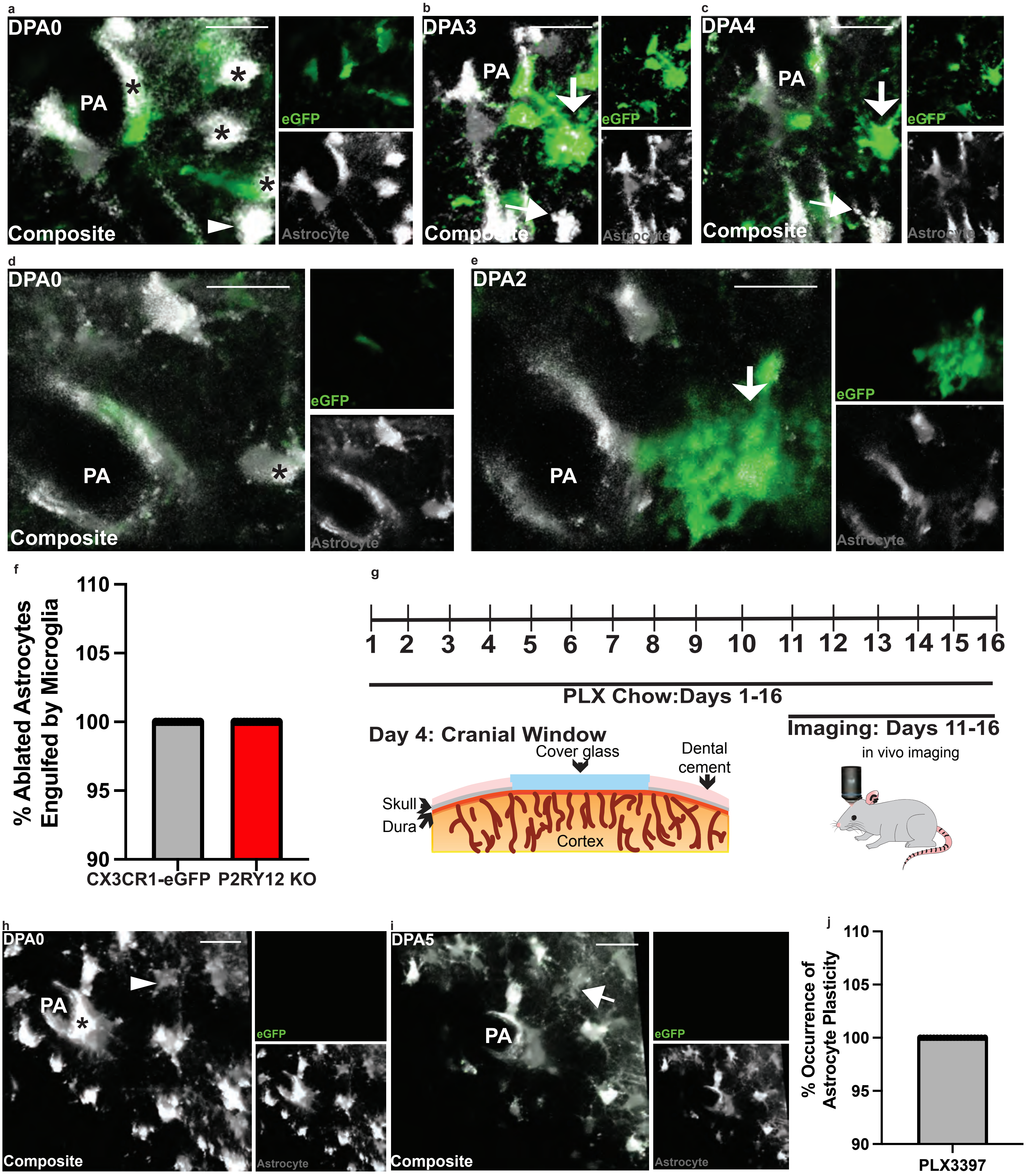

a
DPA0
PA
Composite
eGFP
Astrocyte
b
DPA3
PA
Composite
eGFP
Astrocyte
c
DPA4
PA
Composite
eGFP
Astrocyte
d
DPA0
PA
Composite
eGFP
Astrocyte
e
DPA2
PA
eGFP
Astrocyte
f
% Ablated Astrocytes Engulfed by Microglia
110
105
100
95
90
CX3CR1-eGFP
P2RY12 KO
g
1 2 3 4 5 6 7 8 9 10 11 12 13 14 15 16
PLX Chow:Days 1-16
Day 4: Cranial Window
Cover glass
Dental cement
Skull
Dura
Cortex
Imaging: Days 11-16
in vivo imaging
h
DPA0
PA
Composite
eGFP
Astrocyte
i
DPA5
PA
Composite
eGFP
Astrocyte
j
% Occurrence of Astrocyte Plasticity
110
105
100
95
90
PLX3397

Supplementary Figure 7- P2RY12-independent microglial activation occurs following astrocyte ablation, yet is not necessary for endfoot plasticity to occur- a) Volumetric reconstruction showing the dorsolateral view of astrocytes surrounding a penetrating arteriole (labeled PA). Asterisks indicate ablated astrocytes (grey). Arrow-head indicates the future replacement astrocyte. **b)** Volumetric reconstruction showing the dorsolateral view of the field three days post-ablation (dpa3) exhibiting microglial (green) engulfment of the ablated astrocytes. The arrow with a wide slanted arrow-head indicates the location of microglial engulfment, while the standard arrow indicates the replacement astrocyte. **c)** Volumetric reconstruction showing the dorsolateral view of the same field at dpa4. n=15 fields of view across 3 mice. **d)** and **e)** To test for P2RY12 necessity in ablated astrocyte engulfment, we performed the same experiment in P2RY12 knockout x CX3CR1-eGFP mice. **d)** Volumetric reconstruction showing the dorsolateral view of a field at baseline. Astrocytes surround a penetrating arteriole (PA). A black asterisk indicates the ablated astrocyte. **e)** Volumetric reconstruction showing the dorsolateral view at dpa2. As indicated by the arrow with a wide slanted arrow-head, a microglial cell is visibly engulfing the ablated astrocyte. n=15 fields of view across 3 mice. **f)** Percentage of time ablated astrocytes are engulfed by microglia in CX3CR1-eGFP and P2RY12-KO mice. n=15 astrocytes across 3 mice for both groups. **g)** Schematic showing experimental timeline for microglial elimination studies using PLX3397. **h)** Volumetric reconstruction from the dorsolateral view shows astrocytes surrounding a penetrating arteriole (PA). Ablated astrocytes are indicated by asterisks. Arrow-head indicates future replacement astrocyte. Note the lack of signal in the eGFP channel at baseline, signifying absence of microglia. **i)** Volumetric reconstruction from the dorsolateral view of the field at dpa5, showing microglial engulfment of the ablated astrocytes. The arrow points to the replacement astrocyte. Note the unabated lack of eGFP signal, indicating the continued absence of microglia. n=20 fields of view across 3 mice. **j)** Percentage of instances in which endfoot plasticity occurred in the absence of microglia. LUTs have been adjusted to emphasize activated microglia as well as replacement astrocytes and associated processes in microglia-depleted mice. Scale bar in all images is 20µm.

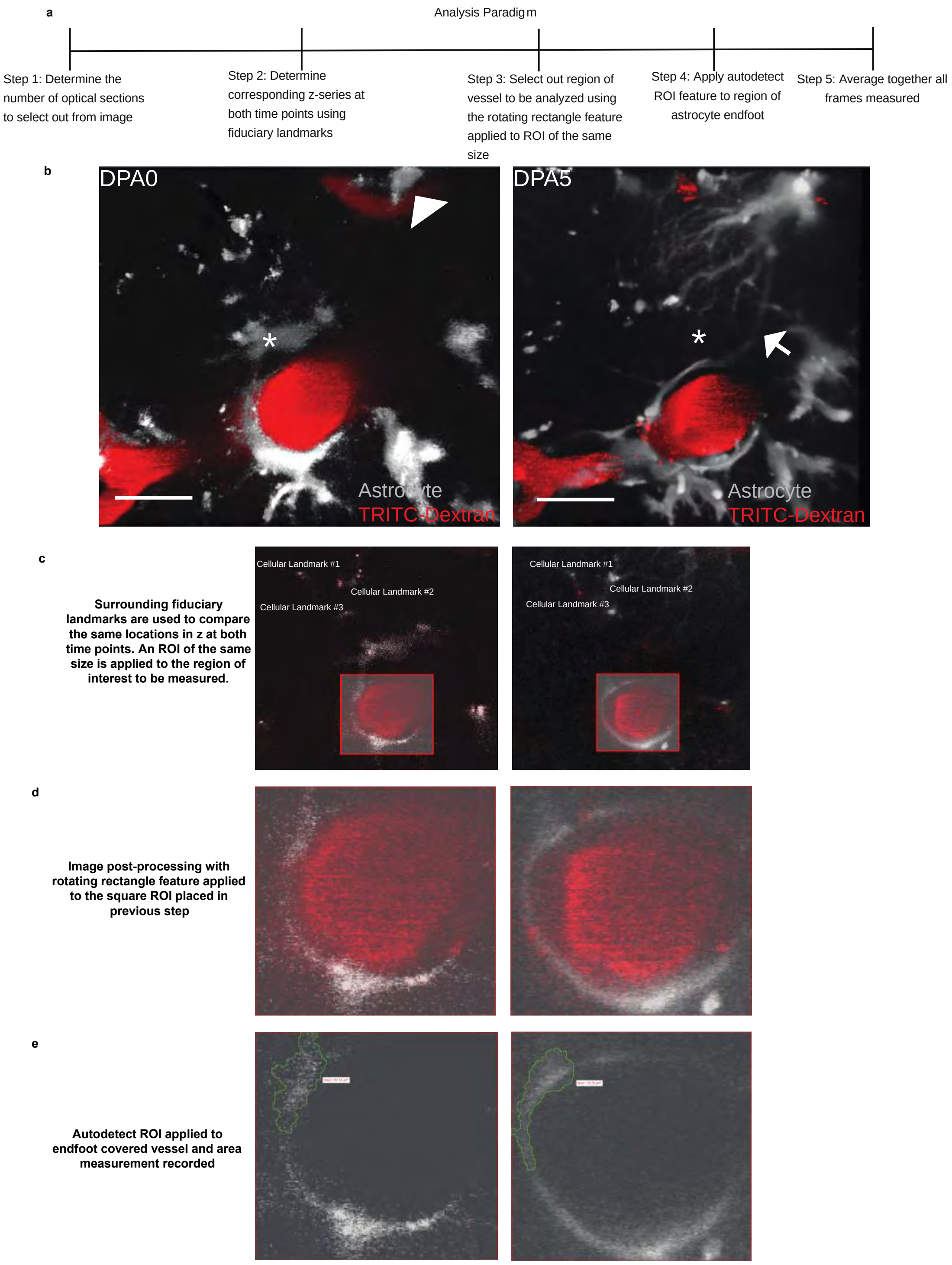

Supplementary Figure 8-Analysis paradigm to quantify area of eGFP+ endfoot lining at penetrating arterioles pre-versus post-ablation of parenchymal-associated astrocytes- a) Graphical representation of the step-wise analysis pipeline used to quantify the area of eGFP+

endfoot areas pre- and post-ablation. The subsequent images in b-d represent this process, where the images in b are volumetric reconstructions showing a field at baseline **(left)** and five days post-ablation **(right)**. Astrocytes are shown in grey and blood vessels in red. **b)** Step one involves identifying locations of endfoot plasticity and determining the number of optical sections to quantify. Sections are selected based on the visibility of the stem portion of the astrocyte. The asterisk indicates the ablated astrocyte and arrow points out the replacement process. The arrow head denotes the replacement astrocyte to-be. n=14 astrocytes across 7 mice. **c)** Surrounding fiduciary landmarks help determine the same or near-same location in the z direction. Once the sections are selected, they are separated out of the native file for further analysis. **d)** A rotating rectangle is then applied to both time points' images, using a reference ROI of the same size for consistency. Note that only endfoot lining immediately around the vessel was chosen for measurements in this analysis. **e)** Finally, the autodetect ROI feature is used to select for, and measure the area of, eGFP+ endfoot coverage. This was repeated across all selected optical sections, and the average of those values is reported in the final analysis for each field of ablation. Scale bar in all images is 20μm.
