# Supplementary material for: Astrocyte plasticity in mice ensures continued endfoot coverage of cerebral blood vessels following injury and declines with age
Source: Nat Commun. 2022 Apr 4;13:1794. doi: 10.1038/s41467-022-29475-2 (PMC8980042; doi:10.1038/s41467-022-29475-2)
Supplement: Supplementary file 3 — Description of Additional Supplementary Files [file 41467_2022_29475_MOESM3_ESM.pdf]

Description of Additional Supplementary Files

Supplementary Movie 1: Movie shows in vivo time lapse video of a GCaMP5G-labelled replacement astrocyte (shown in Figure 2a-b) before and after laser-induced rises in astrocyte intracellular calcium. The white dot appearing in the middle of the video indicates the time and location of laser activation and white bars indicate the location of vascular response. The change in intracellular calcium (Δf/f) and diameter (Δdiameter) are graphed in real time at the top of the video.

Supplementary Movie 2: Movie shows in vivo time lapse video of a GCaMP5G-labelled original astrocyte (shown in Figure 2c) before and after laser-induced rises in astrocyte intracellular calcium. The white dot appearing in the middle of the video indicates the time and location of laser activation and white bars indicate the location of vascular response. The change in intracellular calcium (Δf/f) and diameter (Δdiameter) are graphed in real time at the top of the video.
