# Supplementary material for: Astrocyte plasticity in mice ensures continued endfoot coverage of cerebral blood vessels following injury and declines with age
Source: Nat Commun. 2022 Apr 4;13:1794. doi: 10.1038/s41467-022-29475-2 (PMC8980042; doi:10.1038/s41467-022-29475-2)
Supplement: Supplementary file 6 — Reporting summary [file 41467_2022_29475_MOESM6_ESM.pdf]

nature portfolio

Corresponding author(s): Harald Sontheimer

Last updated by author(s): 2022-Mar-03

# Reporting Summary

Nature Portfolio wishes to improve the reproducibility of the work that we publish. This form provides structure for consistency and transparency in reporting. For further information on Nature Portfolio policies, see our Editorial Policies and the Editorial Policy Checklist.

## Statistics

For all statistical analyses, confirm that the following items are present in the figure legend, table legend, main text, or Methods section.

| n/a | Confirmed | |
|---|---|---|
| ☐ | ☒ | The exact sample size (*n*) for each experimental group/condition, given as a discrete number and unit of measurement |
| ☐ | ☒ | A statement on whether measurements were taken from distinct samples or whether the same sample was measured repeatedly |
| ☐ | ☒ | The statistical test(s) used AND whether they are one- or two-sided<br>*Only common tests should be described solely by name; describe more complex techniques in the Methods section.* |
| ☒ | ☐ | A description of all covariates tested |
| ☐ | ☒ | A description of any assumptions or corrections, such as tests of normality and adjustment for multiple comparisons |
| ☐ | ☒ | A full description of the statistical parameters including central tendency (e.g. means) or other basic estimates (e.g. regression coefficient) AND variation (e.g. standard deviation) or associated estimates of uncertainty (e.g. confidence intervals) |
| ☐ | ☒ | For null hypothesis testing, the test statistic (e.g. *F*, *t*, *r*) with confidence intervals, effect sizes, degrees of freedom and *P* value noted<br>*Give P values as exact values whenever suitable.* |
| ☒ | ☐ | For Bayesian analysis, information on the choice of priors and Markov chain Monte Carlo settings |
| ☒ | ☐ | For hierarchical and complex designs, identification of the appropriate level for tests and full reporting of outcomes |
| ☒ | ☐ | Estimates of effect sizes (e.g. Cohen's *d*, Pearson's *r*), indicating how they were calculated |

*Our web collection on statistics for biologists contains articles on many of the points above.*

## Software and code

Policy information about availability of computer code

| | |
|---|---|
| Data collection | Olympus Fluoview FV315-SW version 2.5.1.228 (Powered by H-PF Version 2.13.2.139) was used for data acquisition. |
| Data analysis | Nikon Imaging Software-Elements AR version 5.02.01 was used to analyze data in this study along with FIJI. Prism 9 was used for all statistical analysis. The mouse graphic used in Figure 1a and elsewhere in this paper was created using Adobe Illustrator. |

For manuscripts utilizing custom algorithms or software that are central to the research but not yet described in published literature, software must be made available to editors and reviewers. We strongly encourage code deposition in a community repository (e.g. GitHub). See the Nature Portfolio guidelines for submitting code & software for further information.

## Data

Policy information about availability of data

All manuscripts must include a data availability statement. This statement should provide the following information, where applicable:

- Accession codes, unique identifiers, or web links for publicly available datasets
- A description of any restrictions on data availability
- For clinical datasets or third party data, please ensure that the statement adheres to our policy

All data will be made upon request to Harald Sontheimer. Many of the reported studies involve comparisons of the same field at different time points. In every instance, a small number of cells were ablated, so one cannot simply look at pre- versus post-ablation time points and assume the same treatment for all cells shown in that field. While we have an ablation map marking all cells ablated at baseline, the exporting process from our software sometimes results in errors to the ROIs that indicate ablated cells. Due to the aforementioned reasons, we feel it is best to provide data on a case-by-case basis rather than on a public repository. We are happy to do so for interested parties.

# Field-specific reporting

Please select the one below that is the best fit for your research. If you are not sure, read the appropriate sections before making your selection.

[x] Life sciences [ ] Behavioural & social sciences [ ] Ecological, evolutionary & environmental sciences

For a reference copy of the document with all sections, see nature.com/documents/nr-reporting-summary-flat.pdf

# Life sciences study design

All studies must disclose on these points even when the disclosure is negative.

| | |
|---|---|
| Sample size | Sample sizes were chosen based off previous studies measuring the same readout. For example, Berthiaume et al 2018 for BBB studies (6 vessels/3 mice). Given that these sample sizes were previously published, and the paired-end nature (before and after in time in the same animal) of most of our analyses, we feel our samples sizes are sufficient. |
| Data exclusions | Only failed ablation attempts were excluded from analysis, but this was rare in our hands. |
| Replication | We have utilized both pharmacological and transgenic means to address the same question regarding molecular mechanism . We further have employed the same assay following ablation and pharmacological studies to measure BBB integrity. Findings BBB studies, findings were replicated four times (young mice, old mice, 1X AG490 injection, 3X AG490 injection). For AG490 studies exploring astrocyte volume reductions at post-ablation timepoints, findings were replicated twice (1X and 3X AG490 injections). All replications were performed independently of one another. Last, we used two multiphotons in this study, with findings replicated on both systems, which were equipped with the same objective. |
| Randomization | Samples were assigned a number. Numbers were assigned to groups. Google random number generator was then used to assign samples to groups. |
| Blinding | Blinding was performed for data analysis. Samples were assigned a unique code, and those performing analyses were given the code only. Blinding was not possible for data acquisition as the person imaging at post-ablation time points needed to know where cells were ablated at pre-ablation baseline timepoints. This necessity of the study prevented blinding in the acquisition process. |

# Reporting for specific materials, systems and methods

We require information from authors about some types of materials, experimental systems and methods used in many studies. Here, indicate whether each material, system or method listed is relevant to your study. If you are not sure if a list item applies to your research, read the appropriate section before selecting a response.

## Materials & experimental systems

| n/a | Involved in the study | |
|---|---|---|
| [x] | [ ] | Antibodies |
| [x] | [ ] | Eukaryotic cell lines |
| [x] | [ ] | Palaeontology and archaeology |
| [ ] | [x] | Animals and other organisms |
| [x] | [ ] | Human research participants |
| [x] | [ ] | Clinical data |
| [x] | [ ] | Dual use research of concern |

## Methods

| n/a | Involved in the study | |
|---|---|---|
| [x] | [ ] | ChIP-seq |
| [x] | [ ] | Flow cytometry |
| [x] | [ ] | MRI-based neuroimaging |

## Animals and other organisms

Policy information about studies involving animals; ARRIVE guidelines recommended for reporting animal research

| | |
|---|---|
| Laboratory animals | For all studies, both male and female mice were used. Young mice were aged 2-4months. Aged mice were 12-months of age. All mice were housed under controlled temperature, humidity, and light (12:12 h light-dark cycle) with food and water readily available ad libitum. The following transgenic lines were used. Swiss Webster-Aldhlll-eGFP bacterial artificial chromosome transgenic mice (generated by the GENSAT project); NG2-dsRedBAC mice (Jackson Labs number 008241) were crossed with Aldhlll-eGFP mice, and mice homozygous for both transgenes were maintained as a colony. Aldhlll-cre (Jackson Labs number 023748) mice were crossed with CAG-GCaMPSG-tdTomato (Jackson Labs number 024477) mice. Aldhlll-cre/ERT2 mice (Jackson lab number 031008) were crossed with STAT3-loxP mice (Hermann et al., 200833, Takeda et al., 1998). CX3CR1-eGFP and P2RY12KO mice were a generous gift from Ukpong B. Eyo . |
| Wild animals | No wild animals were used in the study. |
| Field-collected samples | No field-collected samples were used in the study. |
| Ethics oversight | All studies were approved by the Institutional Animal Care and Use Committees of the University of Virginia and Virginia Tech, and |

were conducted in compliance with the National Institutes of Health's 'Guide for the Care and Use of Laboratory Animals.'

Note that full information on the approval of the study protocol must also be provided in the manuscript.
